# To Evaluate Effect of Airborne Particle Abrasion using Different Abrasives Particles and Compare Two Commercial Available Zirconia on Flexural Strength on Heat Treatment

**Published:** 2017-06

**Authors:** Hari A. Prasad, Naveed Pasha, Mohammed Hilal, G. S. Amarnath, Vinaya Kundapur, M Anand, Sumeet Singh

**Affiliations:** 1 Reader, Department of Prosthodontics, MR Ambedker Dental College & Hospital, Bangalore, India;; 2 Post graduate student, Department of Prosthodontics, MR Ambedkar dental college & hospital, Bangalore, India;; 3 Reader, Department of Prosthodontics, MR Ambedker Dental College & Hospital, Bangalore, India;; 4 Prof & HOD, Department of Prosthodontics, MR Ambedkar dental college & hospital Bangalore, India;; 5 Senior lecturer, Department of Prosthodontics, MR Ambedkar dental college & hospital, Bangalore, India;; 6 Reader, Department of Prosthodontics, MR Ambedkar dental college & hospital, Bangalore, India;; 7 Post graduate student, Department of prosthodontics, MR Ambedkar dental college & hospital, Bangalore, India

**Keywords:** Biaxial Flexural strength, Airborne abrasion, Heat treatment, Silica coated Alumina, Ceramill (AMANNGIRRBACH), ZR-White (UPCERA)

## Abstract

**Background and objective:**

The popularity of ceramic restorations can be attributed to its life-like appearance, durability and biocompatibility and therefore ceramic restorations have been widely used for anterior and posterior teeth. Ceramic restorations have esthetic and biocompatible advantages but low fracture resistance. Since it has high flexural strength and fracture resistance, yttria-stabilized tetragonal zirconia polycrystal (Y-TZP) is the dental material most commonly used for the core of ceramic crowns and fixed dental prosthesis. In spite of improved mechanical properties, acceptable marginal adaptation and biocompatibility the whitish opacity of zirconia is an obvious esthetic disadvantage. The zirconia framework is often veneered with conventional feldspathic porcelain to achieve a natural appearance. However it is difficult to achieve sufficient bond strength between zirconia and the veneering material. Achieving sufficient bond strength between the veneering ceramic and the zirconia core is a major challenge in the long term clinical success of veneered zirconia restorations. The main objective of this study is to evaluate the effect of different surface treatments on the fracture strength of the two commercially available Zirconia namely Ceramill and ZR-White (AMANNGIRRBACH and UPCERA) respectively.

**Method:**

Two commercially available pre-sinteredyttrium stabilized Zirconia blanks (ZR-White and Ceramill) from AMANNGIRRBACH and UPCERA respectively are used to produce the disc shaped specimens of size (15.2 ± 0.03 mm in diameter and 1.2 ± 0.03 mm thick) from each Zirconia blank. All disc shaped specimens are heated at 1200°C in a furnace for 2 hours to form homogenous tetragonal ZrO_2_. The dimensions of the specimens are measured with a digital caliper (aerospace). The thickness and diameter of each specimen are calculated as the means of 3 measurements made at random sites. 80 discs from each Zirconia blank are divided into ten groups of 8 specimens each. Heat treatment after airborne-particle abrasion using 50 µm Al_2_O_3_ particles and 50 µm silica coated Al_2_O_3_ are applied to the upper and lower surfaces of the specimens. Each specimen is held under a pressure of 30 psi for 15 seconds at a direction perpendicular to the surface and at a distance of 30mm with an airborne particle abrasion device for the specimens in the airborne particle abraded groups. Heat treatments were performed at a starting temperature of 500°C, heating rate of 100°c/ min, ending at a temperature of 1000°C and 15 minutes holding time without vacuum for the specimens in the group 4, 5, 9 and 10. Airborne-particle abrasion mimicking the preparation for cementation was applied to the lower surfaces with 50 µm alumina and silica coated alumina particles for the specimens in the groups 6, 7, 8, 9 and 10. The specimens were cleaned for 15 minutes in an ultrasonic bath containing distilled water.

To determine the fracture strength, a disc of 10 mm diameter was used to place 3 hardened steel balls of 3 mm diameter separated each other by 120 degrees (described in the ISO standard 6872 for dental ceramics). Each specimen was centrally placed on this disc. The lower surface mimicking the internal surface of zirconia was the tension side, facing the supporting device testing, while the upper surface mimicking the external surface of the zirconia core was loaded with a flat punch (1 mm in diameter). A universal testing machine was used to perform the test at a cross head speed of 1mm/min. The failure stress was calculated with the equation listed in ISO 6872. The results were then statistically analyzed. A post hoc test was used for pair wise comparisons.

**Result:**

The mean fracture strength of commercially available Zirconia Ceramill (AMANNGIRBACH) showed a significant higher value compared to the ZR-White (UPCERA) Zirconia (*P*<0.001), Airborne abrasion treatment to the specimens showed a significant difference between the abraded groups and the control group (*P*<0.001); further AMANNGIRRBACH specimens gave a higher value compare to the UPCERA specimens. The study also revealed that the heat treatment of the specimens gave significant value (*P*<0.001) compared to the control group, but heat treatment following the air abrasion reduces the fracture strength of the sample than the air abraded group.

**Conclusion:**

Within the limitation of this study, it is concluded that, the *in vitro* fracture strength of Zirconia specimens treated with an airborne abrasion both on the veneering surface (50 µm silica coated Al2O3) and the cementing surface (50 µm Al_2_O_3_) was significantly higher than the heat treated and the control group.

Airborne particle abrasion followed by the heat treatment reduces the fracture strength of the specimen than that ofthe group treated only by the air abrasives.

The fracture strength of a commercially available Ceramill (AMANNGIRRBACH) is greater than that of a Zirconia from ZR-White (UPCERA) variety.

## INTRODUCTION

The focus of dentistry in the present times is not only on the prevention and treatment of disease but also on meeting the demands for better esthetics. Accordingly, several treatment options have been proposed to restore the esthetic appearance of the dentition. The esthetics of a dental restoration depends on the chosen material, anatomical form, surface texture, translucency and colour. So to accurately reproduce the appearance of a natural tooth, considering the patterns of reflection and absorption of the light is not an easy task (1).

Ceramics have been widely used in dentistry because of their ability to provide excellent cosmetic results that mimic natural teeth; they exhibit good mechanical strength when subjected to masticatory efforts. This rapid development of ceramic systems and processing enabled the treatment of teeth in both the anterior and posterior areas with primary objective of properly restoring form, function and esthetic excellence without the presence of metal. In 1774, the French pharmacist, Alex Duchateau and a Dentist named Nicholas Dubois De Chamant, managed to fabricate the first dental porcelain composition based on “green traditional” porcelain. However these prostheses were abandoned due to their high opacity. In 1838, Elias Wildman made porcelain that was more translucent close to the teeth (2). But, this feldspathic porcelain showed a great mechanical fragility due to its crystalline structure. This lead to the introduction of metal infrastructure associated with ceramics, in order to compensate for the low fracture resistance of the porcelain. This association became known as “metalloceramic prosthesis” and represents a milestone in the technological advancement of dental prostheses. A series of events took place after the introduction of these prostheses; improved technique of ceramic processing, formulations of medium and high fusion porcelain and the introduction of vaccum electric furnaces (3).

The use of metalloceramic prostheses over the last 50 years has minimized the problem with porcelain fragility; however its esthetic potential was limited due to the presence of metal. This metal framework acts as a barrier to the transmission of light, giving the prosthetic dental restoration an unesthetic opaque aspect with the presence of darkening at the cervical region of the prosthesis. So to overcome this, Mc Lean and Huges introduced Aluminium oxide (Al_2_O_3_) as a reinforcing phase in dental porcelain in 1965 (2). The first ceramic infrastructures made of Alumina were obtained by a process known as craft ceramic infiltration slip casting, where an infrastructure of high density crystal is prepared with a small amount of glass. During sintering, the glass melts and penetrates the infrastructure through capillary action and creates a ceramic surface with a very low porosity and high flexural strength. Three infiltrating systems were developed reinforcing with alumina and strengthening with Magnesia or Zirconia. The flexural strength varies according to the reinforcement used; Alumina (400 Mpa), Magnesia (300 Mpa) and Zirconia (750 Mpa). However, both the concentration of Alumina and Zirconia shown in these ceramic system imparts improvement of the optical qualities of the restoration, due to its high opacity (4, 5). Additionally, the porosity incorporated during the manufacture of the infrastructure can affect the strength of these restorations (6).

Parallel to the introduction of infiltrated ceramics, glass ceramics have been improved to be applied in the vacuum injection technique, similar to the traditional technique of metal casting. Two glass ceramic compositions were introduced Leucite based (IPS-empress, Ivoclarvivadent) and Lithium disilicate based. In the first composition, Leucite is responsible for strengthening the ceramic when compared to feldspathic porcelain. In second the composition, the high content of crystalline Lithium disilicate enables a volume increase of up to 60% and high flexural strength (300 Mpa), allowing the design of fixed partial prosthesis of upto three units (4, 5). Due to an increasing interest in esthetics and concerns about toxic and allergic reactions to certain alloys, patients and dentists have been looking for metal free tooth coloured restorations. Therefore, the development of new high strength dental ceramics, which appears to be less brittle, less limited in their tensile strength and less subject to time dependant stress failure, has dominated in the later part of 20th century.

Zirconia has been known as a gem since ancient times. Zirconium (Zr) is a chemical element and its name originates from the Persian “Zar-Gun” meaning golden in color. Zr belongs to the transitional metals and its atomic number is 40 and atomic mass 91.224g·mol^-1^. The melting temperature of Zr is 1855°C and the boiling temperature is 4371°C. Zirconia was originally discovered by the chemist Martin Heinrich Klaproth in Germany in 1789 and was isolated by the Swedish chemist Jons Jacob Berzelius in 1824 (7). “Zirconia ceramics or by night, all cats are grey.” It is this transformation of the ZrO_2_ material from tetragonal to monoclinic phase that differentiates it from other ceramic materials (8).

“The addition of oxides such as Calcium, Magnesium, yttrium to the Zirconia can create partially stabilized zirconia (PSZ) which is stable at room temperature. PSZ usually consists of all three phases (7). The cubic phase is not a favourable phase because it accumulates the yttrium and the remaining tetragonal phase is not stable (9). It is also possible to create PSZ at room temperature consisting of primarily the tetragonal phase (Tetragonal Zirconia Polycrystalline-TZP) by the addition of 2-3% mol yttrium oxide (7). The ability to retain the tetragonal phase at room temperature provides very favourable mechanical properties. Under stress, i.e. at the tip of a crack, the 3Y-TZP undergoes a phase transformation from tetragonal to monoclinic phase. This phase transformation results in a 3-4% volumetric expansion inducing a compressive stress in the area of the crack and theoretically prevents crack propagation (7). This strengthening mechanism is known as transformation toughening and makes ZrO_2_ much stronger compared to all other ceramic materials.

Inspite of improved mechanical properties, acceptable marginaladaptation and biocompatibility the whitishopacity of zirconia is an obvious esthetic disadvantage. The zirconia framework is often veneered with conventional feldspathic porcelain to achieveanatural appearance. However, it is difficult to achieve sufficient bond strength between zirconia and the veneering material. Achieving sufficient bond strength between the veneering ceramic and the zirconia core is a major challenge in the long term clinical success of veneered zirconia restorations. After the fabrication of the zirconia core, surface treatments suchas airborne particle abrasion are typically performed on the zirconia surface before veneering. Heat treatment is recommended after airbornep article abrasion in dental practice in order to release the compressive stresses initiated by the air borne particle abrasion. The bonding between the zirconia core and the tooth is also important for the clinical success of zirconia restorations. Attempts have been made to create roughened ceramic surface to provide higher bond strength, thus creating a stronger micromechanical interlock. To increase the surface area for micromechanical bonding, airborne particle abrasion has been used. Some reports have suggested that zirconia treated with airborne particle abrasion easily develops surface cracks or fractures, and this may reduce its mechanical strength (10). However, no studies on flexural strength testing after surface treatment of the internal and external surfaces of zirconia have been done. The purpose of this study was to investigate the effect of airborne particle abrasion using different types of alumina abrasive particles and heat treatment on the flexural strength of the two commercially available zirconia before veneering and cementation.

## REVIEW OF LITERATURE

A radiological study was conducted to determine the transformation zone size in ceramics containing tetragonal ZrO_2_. Results showed the greatest significance for the mechanical properties is the tetragonal (t) monoclinic (m) phase transformation that occurs by a diffusionless shear process at near sonic velocities, similar to those of martensite formation in quenched steel. The results obtained on plane fracture surfaces of various composites were correlated to mechanical property data and transmission electron microscopy observations (TEM) and were found to be in good agreement. This indicates that the method presented is a useful tool for estimating stress-induced transformation zone sizes and for checking the effectiveness of tetragonal particles introduced in a ceramic matrix (11).

A study was conducted where X-ray diffraction data were presented in support of the existence of ferroelasticity in tetragonal zirconia. Reorientation of ferroelastic domains by externally applied stressis proposed as a toughening mechanism. Toughening by this mechanism can occur in addition to transformation toughening. It explains high toughness of some zirconia as which exhibit no transformation to the monoclinic form. Zirconia ceramics toughened by the ferroelastic mechanism have the potential to retain high toughness at elevated temperature unlike transformation toughening (12).

A study was conducted to demonstrate that quantitative fractography can be used to study failed aluminous and glass-ceramic central porcelains. Fracture surfaces of DICOR and Vitadur-N core porcelain modulus-of-rupture bars were studied to identify fracture mirror features useful in locating the source of fracture and calculating the stress at fracture in clinically failed restorations. The morphology of fracture surfaces results from events related to the initiation and propagation of the crack front during failure. Modulus-of-rupture testing was performed in four-point bending. Fracture surfaces were studied by scanning electron microscopy. The mean fracture stress for the Vitadur-N porcelain was found to be higher than the DICOR surface (13).

A study was conducted to evaluate the effects of sand blasting and coating techniques on volume loss, surface morphology, and surface composition of a glass infiltrated alumina ceramic. Volume loss through sandblasting was 36 times less for In-Ceram ceramic compared with a feldspathic glass ceramic (IPS-Empress), and sandblasting of In-Ceram ceramic did not change its surface composition. Sand blasting of all ceramic clinical restorations with feldspathic glass materials should be avoided, but for In-Ceram ceramic the volume loss was within an acceptable range and similar to that of noble metals (14).

A hypothesis was formulated that adhesive bonding methods suitable for glass-infiltrated alumina ceramic can also be used to bond successfully to YPSZ. To prove this hypothesis, bonding methods suitable for alumina ceramic were used on YPSZ and the tensile bond strength and their durability evaluated *in vitro*. The results show that the durable bond to alumina ceramic is achieved with the same resin composites and tribochemicalsilica coating of YPSZ did not result in a durable resin bond as it does on glass-infiltrated alumina ceramic (15).

An *in vitro* study was conducted to evaluate the effect of grinding and sandblasting on the microstructure, biaxial flexural strength and reliability of two yttria stabilized tetragonal zirconia(Y-TZP) ceramics. There was no difference in mean strength between the sintered fine and coarse grained Y-TZP. Significant differences were found between the control group and ground fine grained material for both wet and dry grinding. Sandblasting significantly increases the strength in fine and coarse grained material. The highest amount of monoclinic phase and largest transformed zone depth (TZD) was found after sandblasting. This signifies that sandblasting may provide a powerful technique for strengthening Y-TZP in clinical practice. In contrast, grinding may lead to substantial strength degradation and reduced reliability of pre-fabricated zirconia elements (16).

A study was done to compare the fracture strength of endodontically treated, crowned maxillary incisors with limited ferrule length and different post-and-core systems after fatigue loading. Sixty-four caries-free, human maxillary central incisors were divided into 4 groups. After root canal treatment, Group 1 was restored with titanium posts and composite cores, Group 2 with zirconia posts and composite cores, and Group 3 with zirconia posts and heat-pressed ceramic cores. Teeth restored with cast-on gold posts and cores served as the controls (Group 4). The results suggest that zirconia posts with ceramic cores can be recommended as an alternative to cast posts and cores. If a chairside procedure is preferred, zirconia or titanium posts with composite cores can be used (17).

A study was conducted to evaluate the effect of three different surface treatments on the bond strength of four different luting cements—three bis-GMA–based resin cements and a compomer cement—to In-Ceram. Eight In- Ceram samples were used for each experimental group. The samples were randomly assigned three treatment conditions: 1) etching for 90 seconds with 5% hydrofluoric acid gel, 2) sandblasting (110-μm Al_2_O_3_), and 3) tribochemicalsilica coating. All samples were silanated following the surface treatment. The luting cements were bonded to In- Ceram specimens using Teflon tubes. All samples were thermocycled for 5,000 cycles altering between 5 and 55°C with 30-second dwell times. The shear bond strength values were measured in a universal testing machine with a cross-head speed of 1 mm/min. The result showed that, Shear bond strength of compomer cement following tribochemicalsilica coating was significantly lower in comparison to resin cements. Luting of In-Ceram with various resins provided varying degrees of bondstrengths that were significantly increased by the tribochemicalsilica-coating system (18).

An *in vitro* study has been made on the effects of sandblasting on the strength of Y-TZP and alumina ceramic layer joined to polymeric substrate and loaded at the surface by a spherical indenter, in simulation of occlusal contact in ceramic crowns on tooth dentin. The sandblast treatment is applied to the ceramic interior surface before bonding to the surface, as in common dental practice. Specimens with polished surface are used as control. Tests are conducted with monotonically increasing (dynamic) and sinusoidal (cyclic) loading on the spherical indenter, up to the point of initiation of radial fracture at the ceramic bottom surface immediately below the contact. For the polished specimen, data from the dynamic and cyclic test overlap, consistent with a dominant slow growth mode of fatigue. Strength of the sandblasted specimens shows significant reduction in the dynamic and cyclic tests, indicating of larger starting flaws. However shiftis considerably greater in the cyclic data, suggesting some mechanically assisted growth of the sandblast flaws (19).

A study was done to investigate the strength of the substructure and veneering porcelain interface in all-ceramic systems. The all-ceramic systems tested with their respective veneering porcelains were IPS-Empress2 with Eris (IE), Procera AllCeram with AllCeram (PA), Procera AllZircon with CZR (PZ), and DC-Zircon with Vita D (DC). The veneering porcelain recommended by the manufacturer for each material was fired to the ceramic core. A metal ceramic (MC) combination was tested as a control group. Study showed that the bond strengths of 3 of the tested all-ceramic (IE, PZ, and DC) were not significantly different from the control (MC) group (20).

A study was done to assess the influence of sandblasting, grinding, grinding orientation, polishing and heat treatment on the flexural strength of a yittria stabilized tetragonal Zirconia. The specimens (160 beams) were equally divided into four groups according to the surface treatment (sandblasted, polished, ground parallel to the tensile axis, ground perpendicular). Twenty specimens from each group underwent heat treatment under the firing conditions used to fire a layer of porcelain and glaze. After treatment, the three-point flexure test was used to calculate the flexural strength and X-ray diffraction analysis was used to estimate.The study suggests that sandblasting and grinding may be recommended to increase the strength of dental Y-TZP, provided they are not followed by heat treatment. Fine polishing may remove the layer of compressive stresses and therefore, lower the mean flexural strength (21).

A study was conducted to evaluated the effect of three surface conditioning methods on the microtensile bond strength of resin cement to a glass-infiltrated zirconia-reinforced alumina-based core ceramic. Thirty blocks (5 × 5 × 4 mm) of In-Ceram Zirconia ceramics (In-CeramZirconia-INC-ZR, VITA) were fabricated according to the manufacturer’s instructions and duplicated in resin composite. The specimens were polished and assigned to one of the following three treatment conditions: 1) Airborne particle abrasion with 110 mm alumina particles silanization, 2) Silica coating with 110 mm Silica particles (Rocatec Pre and Plus, 3M ESPE) C silanization, 3) Silica coating with 30 mm SiO_2_ particles (CoJet, 3M ESPE) C silanization. The results showed that Silica coating with silanization either using 110 mm SiO_2_ or 30 mm SiO_2_ particles increased the bond strength of the resin cementto the zirconia-based ceramic significantly compared to that of airborne particle abrasion with 110-mm Al_2_O_3_ (22).

A study was conducted to evaluate the effect of two surface conditioning methods on the microtensile bond strength of a resin cement to three high-strength core ceramics: high alumina-based (In-Ceram Alumina, Procera AllCeram) and zirconia-reinforced alumina-based (In-Ceram Zirconia) ceramics. Ten blocks (5 × 6 × 8 mm) of In-Ceram Alumina (AL), In-Ceram Zirconia (ZR), and Procera (PR) ceramics were fabricated according to each manufacturer’s instructions and duplicated in composite. The specimens were assigned to one of the two following treatment conditions: 1) airborne particle abrasion with 110-μm Al_2_O_3_ particles + silanization, 2) silica coating with 30 SiO_2_ particles (CoJet, 3M ESPE) + silanization. Conditioning the high-strength ceramic surfaces with silica coating and silanization provided higher bond strengths of the resin cement than with airborne particle abrasion with 110-μm Al_2_O_3_ and silanization (23).

A study was conducted to evaluate the effect of sandblasting, grinding, grinding orientation, polishing and heat treatment on the flexural strength of a yittria stabilized tetragonal zirconia polycrystals ceramic (Y-TPZ). The specimens (160 beams) were equally divided into four groups according to the surface treatment (sandblasted, polished, ground parallel to the tensile axis, ground perpendicular). Twenty specimens from each group underwent heat treatment under the firing conditions used to fire a layer of porcelain and glaze. After treatment, the three-point flexure test was used to calculate the flexural strength and X-ray diffraction analysis was used to estimate the relative amount of monoclinic phase. study suggests that sandblasting and grinding may be recommended to increase the strength of dental Y-TZP, provided they are not followed by heat treatment. Fine polishing may remove the layer of compressive stresses and therefore, lower the mean flexural strength (24).

A study was done to Characterize the microstructure, composition and some physical properties of a glass-infiltrated alumina/zirconia-reinforced ceramic (IZ) and the effect of surface treatment on topography. Ceramic specimens were fabricated according to ISO6872 instructions and polished through 1mm alumina abrasive. Quantitative and qualitative analyses were performed using scanning electron microscopy (SEM), backscattered imaging (BSI), electron dispersive spectroscopy (EDS) and stereology. The elastic modulus and Poisson’s ratio were determined using ultrasonic waves, and the density using a helium pycnometer. The following ceramic surface treatments were used: AP-as-polished; HF-etching with 9.5% hydrofluoric acid for 90 s; SB-sandblasting with 25mm aluminum oxide particles for 15 s and SC-blasting with 30mm aluminum oxide particles modified by silica (silica coating) for 15 s. An optical profilometer was used to examine the surface roughness (Ra) and SEM–EDS were used to measure the amount of silica after all treatments. HF is an inadequate surface treatment for bonding resins to IZ ceramic. Results suggested that treating IZ with either SB or SC produced greater Ra values and the SC showed a significant increase in the surface concentration of silica, which may enhance bonding to resin via silane coupling (25).

A study was done to examine the bonding behavior of a dental YPSZ ceramic. After being subjected to various surface treatments, Denzir (Zirconia ceramic) specimens were bonded to each other using an adhesive resin composite, glass ionomer, or zinc phosphate cement. Bonding strength was then determined by the shearing test. No significant differences were observed between SiC and Al_2_O_3_ blasted specimens. In all surface treatments, the shear bond strength significantly increased in the order of adhesive resin composite cement > glass ionomer cement > zinc phosphate cement (26).

A study was done to assess the effect of different surface treatments on the bond strength of veneering ceramics to zirconia. In a shear test, the influences of polishing, sandblasting, and silica-coating of the zirconia surface on bonding were assessed with five different veneering ceramics. In addition the effect of liner application was examined. With one veneering ceramic, the impact of regeneration firing of zirconia was also evaluated. Findings of this study revealed that bonding between veneering ceramics and zirconia might be based on chemical bonds. On this note, sandblasting was not a necessary surface pre-treatment to enhance bond strength and that regeneration firing was not recommended (27).

A study was conducted to evaluated and compared the effect of three trialkoxysilane coupling agents on the bond strength of a Bis-GMA-based unfilled resin and a dimethacrylate-based resin composite luting cement to a zirconia ceramics (Procera® AllZircon, Nobel Biocare, G¨ oteborg, Sweden). Six square-shaped zirconia specimens were used for each test group, a total of 72 specimens. The specimens in each group were all assigned to air-borne alumina particle abrasion followed by tribochemical silica-coating and silanization with 1 vol% solutions of 3 methacryloyloxy propyl trimethoxysilane, 3 acryloyloxypropyl trimethoxysilane,or 3 isocyanapropyl triethoxy silane in an ethanol–water mixture. The sample stubs were made of a Bis-GMA/MMA/DMAEMA resin or a commercial resin composite luting cement (RelyXTM ARC, 3M ESPE, Seefeld, Germany). They were bonded to the conditioned and silanized silica coated zirconia specimens using polyethylene molds. All specimens were tested at dry and thermo-cycled (6000, 5–55ºC, 30 s) conditions. The shear bond strength of resin stubs to zirconia was measured in a universal testing machine (cross-head speed 1 mm/min). Bonding of the experimental resin and commercial cement to silica coated zirconia is effective with 3 methacryloyloxypropyl trimethoxysilane or 3 acryloyloxypropyl trimethoxysilane, but not with 3-isocyanatopropyl triethoxysilane (28).

A study was done to evaluate the effect of surface conditioning on the microtensile bond strength of zirconium-oxide ceramic to dual-cured resin cements. Eighteen cylinder-shaped zirconium-oxide ceramic blocks (Cercon® Zirconia, Dentsply) were treated as follows: 1) Sandblasting with 125 μm aluminum-oxide (Al_2_O_3_) particles; 2) tribochemical silica coating using 50 μm Al_2_O_3_ particles modified by silica; 3) no treatment. Each ceramic cylinder was duplicated in composite resin (TetricEvoCeram, Ivoclar-Vivadent) using a silicon mold. Composite cylinders were bonded to conditioned ceramics using: 1) Calibra (Densply Caulk); 2) Clearfil Esthetic Cement (Kuraray); 3) Rely Unicem (3M ESPE). After 24h bonded specimens were cut into microtensile sticks that were loaded in tension until failure. Significant changes in zirconia surface roughness occurred after sandblasting. Bond strength of Clearfil cement to zirconia was significantly higher than that of Rely Unicem and Calibra, regardless of the surface treatment (29).

A review of literature covering all-ceramic materials and systems, with respect to survival, material properties, marginal and internal fit, cementation and bonding, colour and esthetics and provides clinical recommendations for their use was conducted. The literature demonstrates that multiple all-ceramic materials and systems are currently available for clinical use, and there is not a single universal material or system for all clinical situations. Successful application of these materials will depend upon the clinician’s ability to select the appropriate material, manufacturing technique and cementation or bonding procedures, to match intraoral conditions and esthetic requirements (30).

A study was done to assess how ceramic disc thickness and curing mode (light or chemical) affects the polymerization shrinkage of dual-cured resin cements and to evaluate the effect of the ceramic discs on the curing speed of the cements during light exposure. Six commercial resin cements were used. Filler weight contents were determined. Four ceramic discs with thicknesses of 0.5, 1, 2 and 4mm, respectively were made. The results showed that the polymerization shrinkage and filler weight were inversely related. Both the transmitted light intensity and polymerization shrinkage decreased with increasing thickness of ceramic discs (31).

A study was conducted to assess *in vitro* the marginal fit of four-unit fixed partial dentures (FPDs) produced using three different computer aided design/computer aided manufacturing (CAD/CAM) all-ceramic systems before and after porcelain firing cycles and after glaze cycles. Within the limitations of this study, it was concluded that the three zirconium-oxide-based ceramic CAD/CAM systems demonstrated a comparable and acceptable marginal fit; however, the Lava system produced gap measurements statistically smaller than the Everest and Procera systems. The porcelain firing cycles and the glaze cycles did not affect the marginal fit of the zirconium-oxide-based ceramic CAD/CAM systems (32).

A prospective study was conducted to evaluate the clinical outcome of three-unit posterior fixed dental prostheses (FDPs) made of In-Ceram Zirconia. All FDPs were inserted and cemented with glass-ionomer cement. Follow-ups were performed annually. During a mean observation time of 54 months, two FDPs failed (one technical and one biologic failure). Two FDPs debonded and the veneering ceramic fractured in four cases. Three abutment teeth needed endodontic treatment and two additional abutment teeth exhibited secondary caries. Results suggest that posterior three-unit all-ceramic FDPs made from In-Ceram Zirconia may be a viable prosthetic treatment option with an outcome comparable to metal-ceramic FDPs (33).

A study was done to develop a practical method to chemically modify the surface of high strength dental ceramics (i.e. zirconia) to facilitate viable, robust adhesive bonding using commercially available silanes and resin cements. Investigation focused on a novel approach to surface functionalize zirconia with a SiO_2_ “seed” layer that would promote chemical bonding with traditional silanes. Microtensile testing results revealed that zirconia with a surface treatment of 2.6 nm SiO_2_ thick “seed” layer was similar in strength to the porcelain group (control). Mechanical data support that utilizing a gas-phase chloro-silane pretreatment to deposit ultra-thin silica-like seed layers can improve adhesion to zirconia using traditional silanation and bonding techniques (34).

A study was done evaluate the effect of different chemo-mechanical surface treatments ofzirconia ceramic in the attempt to improve its bonding potential. Sintered zirconium oxide ceramic discs (LavaTM Ø10 mm 1mm height) were treated with (n=4): 1) airborne particle abrasion with 125 mm Al_2_O_3_ particles; 2) 9.5% HF acid etching; 3) selective infiltration etching (SIE); 4) experimental hot etching solution applied for 10, 30 and 60 min; 5) no treatment. Ceramic discs surfaces were analyzed by atomic force microscopy (AFM) recording average surface roughness measurements of the substrate. It was concluded that Zirconia conditioning with the experimental hot etching solution may enhance ceramic roughness and improve the surface available for adhesion formation of micromechanical retention (35).

A study was done to evaluate the bonding strength of the porcelain veneer to the zirconia core and to other various metal alloys (high noble metal alloy and base metal alloy).15 rectangular (4 × 4 × 9 mm) specimens each of zirconia (Cercon), base metal alloy (Tillite), high noble metal alloy (Degudent H) were fabricated for the shear bond strength test. The veneering porcelain recommended by the manufacturer for each type of material was fired to the core in thickness of 3mm. After firing, the specimens were embedded in the PTFE mold, placed on a mounting jig, and subjected to shear force in a universal testing machine. Load was applied at a crosshead speed of 0.5 mm/min until fracture. It was concluded that there was a significant difference between the metal ceramic and zirconia ceramic group in shear bond strength. There was no significant difference between the base metal alloy and the high noble metal alloy (36).

The zirconia–resin bond strength was enhanced using novel engineered zirconia primers in combination with selective infiltration etching as a surface pre-treatment. The study was conducted to evaluate the effect of artificial aging on the chemical stability of the established bond and to understand the activation mechanism of the used primer (37).

Selective infiltration etched zirconia discs (Procera; NobelBiocare) were coated with one of four novel engineered zirconia primers containing reactive monomers and were bonded to resin-composite discs (Panavia F2.0). Fourier transform infrared spectroscopy (FTIR) was carried out to examine the chemical activation of zirconia primers from mixing timeand up to 60 min. The bilayered specimens were cut into microbars (1 mm in cross-section area) and zirconia–resin microtensile bond strength (MTBS) was evaluated immediately andafter 90 days of water storage at 37ºC. Scanning electron microscopy (SEM) was used to analyze the fracture surface. The result revealed that, novel engineered zirconia primers produced initially high bond strength values which were significantly reduced after water storage. Long-term bond stability requires developing more stable primers (38).

A study was done to evaluate the shear bond strength between an indirect composite material and zirconium dioxide (zirconia) ceramics after thermocycling. A total of 80 zirconia (Katana) discs were divided into five groups and primed with one of following agents: All Bond 2 Primer B (ABB), Alloy Primer (ALP), AZ Primer (AZP), Estenia Opaque Primer (EOP), and Porcelain Liner M Liquid A (PLA). An indirect composite material (Estenia C&B) was then bonded to the primed zirconia. One-half of the specimens (n=8) in each group were stored in distilled water at 37°C for 24 h, and the remaining eight specimens were thermocycled 5,000 times before shear bond strength testing. In conclusion, the study demonstrated that the use of an acidic functional monomer containing carboxylic anhydride (4-META), phosphonic acid (6-MHPA), or phosphate monomer (MDP) can yield durable bond strength between Estenia C & B indirect composite and Katana Zirconia (39).

A study was done to determine whether the bond of veneering porcelain to a ceramic core in bilayered ceramics was similar to that of the metal ceramic control of well known behaviour. Six groups of nine specimens and the dimensions were 15 mm long and 8mm in diameter at the core, and 2 mm long and 8 mm in diameter for the veneer. The groups were GR. 1 (control group): CrNi alloy/d.SIGN (Ivoclar), GR. 2: IPS e.maxPress/IPS e.maxCeram (Ivoclar), GR. 3: IPS e.maxZirCad/IPS e.maxZirPress(Ivoclar), GR. 4: IPS e.maxZirCad/IPS e.maxCeram (Ivoclar), GR. 5: Lava Frame (3M ESPE)/Lava Ceram (3M ESPE) and GR. 6: Lava Frame (3M ESPE)/IPS e.max Ceram (Ivoclar). A shear strength test was used in all samples with a universal testing machine. The chosen cross head speed was of 0.50 mm/min. The bond strength of group 1 (control) was similar to groups 3 and 5. Group 2, whose core and veneer are both porcelains with a similar chemical composition, with silica as their main component, achieved the best adhesive results between both porcelains. The technique on zirconia cores that showed the higher results was the pressed technique. The lowest results were for the group using porcelains from different manufacturers (40).

A study was conducted for evaluation of the effect of different surface treatments and type of zirconia (white or colored) on shear bond strength (SBS) of zirconia core and its veneering porcelain. Eighty zirconia disks (40 white and 40 colored; 10 mm in diameter and 4 mm thick) were treated with three different mechanical surface conditioning methods (Sandblasting with 110 μm Al_2_O_3_ particle, grinding, sandblasting and liner application). One group had received no treatment. These disks were veneered with 3 mm thick and 5 mm diameter Cercon Ceram Kiss porcelain and SBS test was conducted (cross-head speed = 1 mm/min). Type of zirconia did not have any effect on bond strength between zirconia core and veneer ceramic. Surface treatment had different effects on the SBS of the different zirconia types and grinding dramatically decreased the SBS of white zirconia- porcelain (41).

A study was done to evaluate, the effect of a simple and novel surface conditioning method on the core-veneer bond strength of a zirconia ceramic system. The shear bond strength of a zirconia core ceramic to the corresponding veneering porcelain was tested by the Schmitz-Schulmeyer method. Thirty zirconia core specimens (10 × 5 × 5 mm) were layered with a veneering porcelain (5 × 3 × 3 mm). Three different surface conditioning methods were evaluated: polishing with upto 1200 grit silicon carbide paper under water cooling, airborne-particle abrasion with 110 μm alumina particles, and modification with zirconia powder coating before sintering. It was concluded that modifying the zirconia surface with powder coating could significantly increase the shear bond strength of zirconia to veneering porcelain (42).

A study was to done to compare the effects of three different surface treatments in enhancing porcelain zirconia bonding. Totally, 160 densely sintered zirconia specimens were prepared and randomly divided into four study groups: control (no treatment, Group C), sandblasting (Group S), sandblasting followed by regeneration firing (Group SH), and laser irradiation (pulse mode) on a CO_2_ laser system (Group L). After surface treatment, porcelain powders were veneered on zirconia surface.

Half of the specimens in each group were evaluated without aging (initial shear bond strength – initial SBS), and the other half was tested after being stored in water for one month (aging SBS). The results showed that both sandblasting and laser irradiation increased porcelain zirconia bond strength. The presented new modified laser pre-treatment might be an alternative way to sandblasting for improving zirconia/porcelain integration (43).

In all-ceramic restoration involving a zirconia frame work, surface treatment of the zirconia surface is required to enhance bonding strength with the veneering ceramics and thus preventing chipping. An *in vitro* study was done to investigate the influence of surface roughness and heat treatment of the zirconia and use of liner porcelain on the bond strength between veneering ceramics and zirconia framework. Debonding /crack initiation strength was determined according to ISO 9693. No significant difference was observed among conditions, except with use of a liner under heat treatment. Electron probe microanalysis revealed that components of the veneering ceramics remained on the zirconia surface after debonding, suggesting that fractures occur in the veneering ceramics and that improving the strength of the veneering ceramics themselves might increase the bond strength (44).

Airborne-particle abrasion of the inner and outer surfaces of an yttria-stabilized tetragonal zirconia polycrystal (Y-TZP) core is used in an attempt to enhance the bond strength between the core and the veneering porcelain and to increase the surface area for cementation. An *in vitro* study was done to investigate the effect of airborne particle abrasion and heat treatment on the microstructure, biaxial flexural strength and reliability of Y-TZP zirconia ceramics before veneering and cementation. Forty eight disks of Y-TZP were divided into 6 groups. Three treatments (untreated, air particle abrasion, heat treatment after air abrasion) were applied to the upper surface, and 2 treatments (untreated and airborne-particle abrasion) were applied to the lower surfaces to mimic the preparation for veneering and cementation. Particle sizes of 110 μm alumina were used. Result showed that the group with airborne-particle abraded lower surfaces showed significantly higher flexural strength than the untreated group. SEM images showed rough and irregular surfaces on the airborne particle abraded surface (45).

An *in vitro* study was done to evaluate the effect of air particle abrasion protocols on the biaxial flexural strength, surface characteristics and phase transformation of zirconia after cyclic loading. Disc shaped zirconia specimen (diameter 15 mm; 1.2 mm thick) were submitted to one of the air particle abrasion protocols, a) 50 μm alumina b)110μm silica coated alumina c) 30 μm silica coated alumina for 20 sec at 2.8 barpressure. All specimens were initially cyclic loaded (× 20000, 50 N, 1 Hz) in water at 37ºC and subjected to biaxial flexural strength where the conditioned surface was under tension. Zirconia surface were characterized and roughness was measured with 3D surface profilometer. Phase transformations from tetragonal to monoclinic were determined by spectroscopy. The relative amount of transformed monocliniczirconia (FM) and transformed zone depth (TZD) were measured using X-ray diffraction (XRD) analysis. The data were analysed using ANOVA. Surface roughness was highest with 50 μm alumina (0.261 μm). After all air abrasion protocols, FM increased (15.02%-19.25%) compared to control group (11.12%). TZD also showed increased after air abrasion protocols, (0.83-1.07 μm) compared to control groups (0.59 μm). Air abrasion protocols increase the roughness and monoclinic phase but in turn abrasion with 30μm alumina particles coated with silica has increased the biaxial flexural strength of the tested zirconia (46).

A review of the literature about cementing or luting indirect restorations to tooth structure was done. It focussed on surface preparation of precious metals, non-precious metals, indirect composite materials and all available porcelain materials including feldspathic, leucite reinforced, lithium disilicate, slip cast aluminium oxide, densely sintered aluminium oxide and zirconia prior to luting. Using data from a variety of sources, product categories of materials and various bonding materials and procedures were ranked according to their bond strength and durability (47).

A study was done investigate different surface treatments of the zirconium dioxide ceramic core and find the best, for achieving highest adhesive bonding values to veneering porcelain. The study was primarily designed to investigate the bonding strength of the veneering porcelain to zirconia with *in vitro* Macro shear bond strength test. The specimens with different surface treatment of the zirconia were divided in five groups of twelve according to the treatment of zirconium surface and results showed highest bonding values for specimens treated with Rocatec system and lowest value for those treated with liner (48).

A study was conducted to evaluate the effect of annealing in air on the crack healing behaviour of a machinable dental ceramic. The glass transition temperature and the softening point were determined by dilatometry. The ratio of crack length after annealing to crack length before annealing treatment was calculated for each indentation. Scanning electron microscopy was performed before and after annealing.

Scanning electron microscopy revealed shortening and blunting of the cracks after annealing. Annealing in air significantly reduced the mean crack length of an indented machinable dental ceramic compared to the control group. However, the mean biaxial flexural strength was not significantly affected by an annealing treatment (49).

## MATERIALS AND METHOD

Eighty disks each of commercially available ZR-White (UPCERA) and Ceramill (AMANNGIRRBACH) pre- sintered Zirconia block of size (15.2 ± 0.03 mm in diameter and 1.2 ± 0.03 mm thick) were fabricated. All disc shaped specimens are heated at 1200°C in a furnace for 2 hours to form homogenous tetragonal ZrO_2_. The dimensions of the specimens are measured with a digital caliper (AEROSPACE). The thickness and diameter of each specimen are calculated as the means of 3measurementsmadeatrandomsites.

The specimens were divided into 10 groups of 8 specimens each, and then heat treatment after airborne-particle abrasion and air borne particle abrasion alone were applied to the upper (veneering) and lower (cementing) surfaces of the specimens.

Five treatments mimicking the preparation for the veneering were applied as follows:

Group 1: no surface treatment;

Group 2: airborne particle abrasion with 50 μm alumina on the veneering side;

Group 3: airborne abrasion with 50 μm silica coated alumina on the veneering side;

Group 4: airborne abrasion with 50 μm alumina and heat treatment after airborne particle abrasion;

Group 5: airborne particle abrasion with 50 μm silica coated alumina and heat treatment after air borne abrasion;

For the cementing surface;

Group 6: airborne particle abrasion with 50 μm alumina on the lower (cementing) surface and no treatment on the veneering side;

Group 7: airborne abrasion on the upper (veneering) surface and lower (cementing) surface with 50 μm alumina;

Group 8: airborne abrasion on the upper surface with 50 μm silica coated alumina and lower surface with 50 μm alumina;

Group 9: airborne abrasion on the upper surface with 50 μm alumina followed by Heat treatment and lower surface air-borne abrasion with 50 μm alumina only;

Group 10: airborne abrasion on the upper surface (50 μm silica coated alumina) followed by Heat treatment and lower surface air-borne abrasion with 50 μm alumina.

The air borne particle abrasion used was 50 μm alumina particles under pressure for 15 seconds at 30 psi in a direction perpendicular to the surface with an airborne-particle abrasion device for the specimens in the airborne-particle abraded groups.

Heat treatments were performed at a starting temperature of 500°C, heating rate of 100°C/min, ending temperature of 1000°C and 15 minutes holding time without vacuum for the specimens in the group 4, 5, 9 and 10.

Airborne particle abrasion mimicking the preparation for cementation was applied to the lower surfaces with 50 μm alumina and silica coated alumina particles for the specimens in the groups 6, 7, 8, 9 and 10. The specimens were cleaned for 15 minutes in an ultrasonic bath containing distilled water.

To determine the fracture strength load, a disc of 10mm diameter was used to place 3 hardened steel balls of 3mm diameter separated each other by 120 degrees (described in the ISO standard 6872 for dental ceramics). Each specimen was centrally placed on this disc. The lower surface mimicking the internal surface of zirconia was the tension side, facing the supporting device testing, while the upper surface mimicking the external surface of the zirconia core was loaded with a flat punch (1mm in diameter).A universal testing machine was used to perform the test at a cross head speed of 1mm/min. The failure stress was calculated with the equation listed in ISO 6872.

$$\text{S}=-0.2387\text{P}(\text{X}-\text{Y})/{\text{d}}^{2}$$

$$\text{X}=(1+\text{v})\text{In}{\left({\text{r}}_{2}/{\text{r}}_{3}\right)}^{2}+\{(1-\text{v})/2\}{\left({\text{r}}_{2}/{\text{r}}_{3}\right)}^{2}$$

$$\text{Y}=(1+\text{v})\{1+\text{In}{\left({\text{r}}_{1}/{\text{r}}_{3}\right)}^{2}\}+(1-\text{v}){\left({\text{r}}_{1}/{\text{r}}_{3}\right)}^{2}$$

where, S = the maximum tensile stress(MPa), P = total fracture load(N), d = specimen thickness(mm), v = poisson ratio, r_1_ = radius of the support circle, r_2_ = radius of the loaded area, r_3_ =radius of the specimen thickness at fracture origin.

Similarly the commercially available Zirconia of other variety is tested in the same pattern and compared.

## RESULT

This study was conducted to evaluate the effect of airborne particle abrasion using different types of alumina abrasive particles and heat treatment on the flexural strength of the two commercially available zirconia before veneering and cementation.

Eighty disk seach of two commercially available ZR-White (AMANNGIRRBACH) and Ceramill (UPCERA) Y-TZP were divided into 10 groups of 8 specimens each, and then heat treatment after airborne-particle abrasion and airborne particle abrasion alone were applied to the upper (veneering) and lower (cementing) surfaces of the specimens.

The airborne particle abrasion used was 50µm aluminaand50µm silica coated alumina particles under pressure 30 psi for 15 seconds at a direction perpendicular to the surface with an airborne particle abrasion device for the specimens in the airborne particle abraded groups 2,3,6,7,8,9 and 10.

Heat treatments were performed at a starting temperature of 500°C, heating rate of 100°C/min, ending temperature of 1000°C and 15 minutes holding time without vacuum for the specimens in the group 4, 5, 9 and 10.

The specimens were cleaned for 15 minutes in an ultrasonic bath containing distilled water.

To determine the fracture strength load, a disc of 10mm diameter was used to place 3 hardened steel ballsof3mm diameters separated each other by 120 degrees (described in the ISO standard 6872 for dental ceramics). Each specimen was centrally placed on this disc. The lower surface mimicking the internal surface of zirconia was the tension side, facing the supporting device testing, while the upper surface mimicking the external surface of the zirconia core was loaded with a flat punch (1mm in diameter). A universal testing machine was used to perform the test at a cross head speed of 1mm/min. The failure stress was calculated with the equation listed in ISO 6872.

$$\text{S}=-0.2387\text{P}(\text{X}-\text{Y})/{\text{d}}^{2}$$

$$\text{X}=(1+\text{v})\text{In}{\left({\text{r}}_{2}/{\text{r}}_{3}\right)}^{2}+\{(1-\text{v})/2\}{\left({\text{r}}_{2}/{\text{r}}_{3}\right)}^{2}$$

$$\text{Y}=(1+\text{v})\{1+\text{In}{\left({\text{r}}_{1}/{\text{r}}_{3}\right)}^{2}\}+(1-\text{v}){\left({\text{r}}_{1}/{\text{r}}_{3}\right)}^{2}$$

where, S = the maximum tensile stress (MPa), P = total fracture load (N), d = specimen thickness (mm), v = poisson ratio, r_1_ = radius of the support circle, r_2_ = radius of the loaded area, r_3_ =radius of the specimen thickness at fracture origin.

Similarly, the commercially available Zirconia of other company is tested in the same pattern and compared.

**Figure 1:**
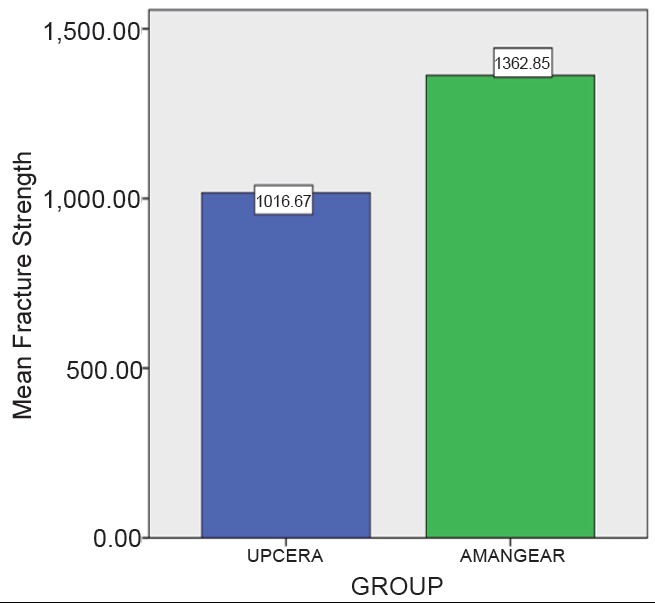
Comparison of mean fracture strength betweentwo commercial companies.

**Figure 2:**
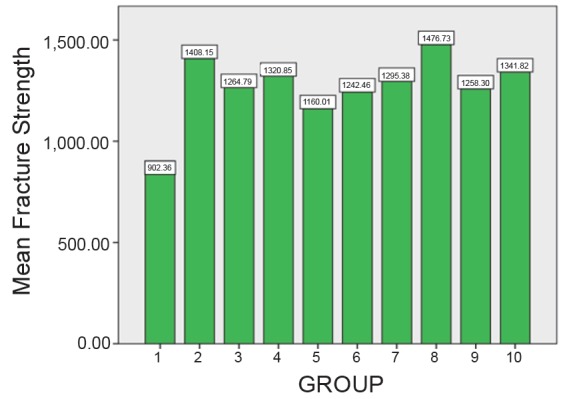
Mean fracture strength on different surface treatments forceramill (amangirrbach) group.

**Figure 3:**
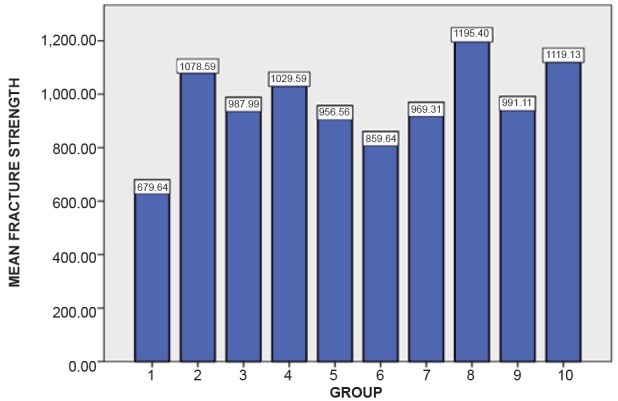
Mean fracture strength on different surfacetreatments forzr-white (upcera) group.

The mean and standard deviation values of different commercially available Zirconia are listed in Table 1 and Table 2. Table 3 and Figure 1 depicts the highest mean fracture strength exhibited by Ceramill (AMANNGIIRRBACH) Zirconia which is statistically significant (*P*<0.001). Table 4 depicts the effect of surface treatment on fracture strength of different group and the control group. Figure 2 depicts the effect of different surface treatment (air abrasive and heat treatment) on the fracture strength of commercially available Ceramill (AMANNGIRRBACH) Zirconia, the result shows highly significant (*P*<0.001) in the group 2, 3, 4, 5, 7, 8, 9, 10 and statistically significant (*P*<0.005) in the group 6. Table 5 depicts the effect of surface treatment on fracture strength of different group and the control group. Figure 3 depicts the effect of different surface treatment (Air abrasive and heat treatment) on the fracture strength of commercially available ZR-WHITE (UPCERA) Zirconia. The result shows highly significant value (*P*<0.001) in group except group 5 which is statistically significant (*P*<0.005).

**Table 1:** The Mean and Standard Deviation for the Fracture Strength of Ceramill (Amanngirrbach)

	n	Mean	Std. Deviation

FRACTURE STRENGTH	80	1362.8500	206.92120

**Table 2:** The mean and standard deviation for the fracture strength of zr-white (upcera)

	n	Mean	Std. Deviation

FRACTURE STRENGTH	80	1016.6670	171.58485

**Table 3:** Comparison of mean fracture strength between two commercial companies

ANOVA			

	AMANNGIRRBACH and UPCERA		

	t	df	Sig. (2-tailed)
FRACTURE STRENGTH	-4.073	18	0.001

**Table 4:** Effect of different types of surface treatment on the zr-white (upcera) group between the surface treated group and the control group

GROUP	n	Mean	P VALUE£

1	8	679.6375	
2	8	1078.5875	<0.001**
1	8	679.6375	
3	8	987.9875	<0.001**
1	8	679.6375	
4	8	1029.5875	<0.001**
1	8	679.6375	
5	8	956.5625	0.001**
1	8	679.6375	
6	8	859.6375	0.015*
1	8	679.6375	
7	8	969.3125	0.001**
1	8	679.6375	
8	8	1195.4000	<0.001**
1	8	679.6375	
9	8	991.1125	<0.001**
1	8	679.6375	
10	8	1119.1250	<0.001**

£ Using t test;

* P value ≤ 0.05 stastistically significant;

** P value ≤ 0.001 highly statistically significant.

**Table 5:** Effect of different types of surface treatment on ceramill (amanngirrbach) group between the surface treated group and the control group

GROUP	n	Mean	P VALUE^£^

1	8	902.3625	<0.001**
2	8	1408.1500	
1	8	902.3625	
3	8	1264.7875	<0.001**
1	8	902.3625	
4	8	1320.8500	<0.001**
1	8	902.3625	
5	8	1160.0125	<0.005*
1	8	902.3625	<0.001**
6	8	1242.4625	
1	8	902.3625	
7	8	1295.3750	<0.001**
1	8	902.3625	0.001**
8	8	1476.7250	
1	8	902.3625	
9	8	1258.3000	<0.001**
1	8	902.3625	<0.001**
10	8	1341.8250	

£ Using t test;

* P value ≤ 0.05 stastistically significant;

** P value ≤ 0.001 highly statistically significant.

Table 6 and Table 8 Depicts the analysis of varience of both ZR White (UPCERA) & cadmill (AMANNGIRRBACH) zirconia with in the group of heat treatment air abrasion group and also between the two surface treated group the results show highly significant value (*P*<0.001) between the heat treated and the air abrasion group.

**Table 6:** Effect of different types of surface treatment (within the heat treated/ air abrasion and between the heat treated—air abrasion) on the zr-white (upcera) group of zirconia anova

	Sum of Squares	df	Mean Square	F	Sig.

Between Groups	960477.176	2	480238.588	20.916	0.000
Within Groups	1767949.122	77	22960.378		
Total	2728426.298	79			

**Table 8:** Effect of different types of surface treatment (within the heat treated / air abrasion and between the heat treated-air abrasion) on the ceramill (amanngirrbach)

GROUP ANOVA CERAMILL (AMANNGIRRBACH)					
	Sum of Squares	df	Mean Square	F	Sig.

Between Groups	890480.048	2	445240.024	11.545	0.000
Within Groups	2969532.154	77	38565.353		
Total	3860012.202	79			

Table 7 and Table 9 depicts the multiple comparisons using post hoc test between the heat treated air abrasive and the control group. The results showed the significant value (*P*<0.001) between the surface treated group and the control group of ZR White (UPCERA) Zirconia.

**Table 7:** Post hoc scheffe test between different surface treatment and the control group of zr-white (upcera)

Tukey HSD				

(A) GROUP	(B) GROUP	Mean Difference (I-J)	Std. Error	Sig.

HEAT TX	-AIR ABRASION	-33.72500	37.88171	0.648
	-CONTROL	254.45938*	46.39542	0.000
AIR ABRASION	-CONTROL	288.18438*	46.39542	0.000

* The mean difference is significant at the 0.05 level.

**Table 9:** Post hoc scheffe test between different surface treatment and the control group of ceramill (amanngirrbach)

Tukey HSD				
(A) GROUP	(B) GROUP	Mean Difference (I-J)	Std. Error	Sig.

HEAT TX	-AIR ABRASION	-91.01250	49.09516	0.159
	-CONTROL	197.83438*	60.12904	0.004
AIR ABRASION	-CONTROL	288.84687*	60.12904	0.000

* The mean difference is significant at the 0.05 level.

**Figure 4:**
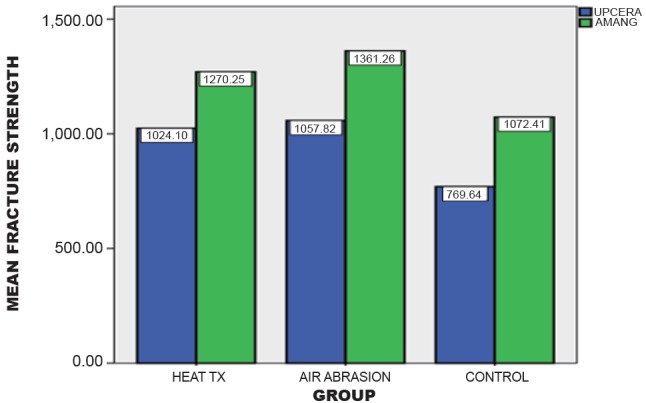
Effect of different surface treatments on the 2 groups.

Ceramill (AMANNGIRRBACH) showed highly significant value (*P*<0.001) between the air abrasion and the control group. Figure 4 depicts the descriptive values of different surface treatments and its effect on fracture strength of two commercially available Zirconia and the control group. The study showed highly significant value in the air abrasive group of Ceramill (AMANNGIIRBACH) than the air abraded ZR White (UPCERA) Zirconia.

## DISCUSSION

The esthetics of a dental restoration depends on the material, anatomical form, surface texture, translucency and colour. So to accurately reproduce the appearance of a natural tooth, considering the patterns of reflection and absorption of the light is not an easy task (1).

Ceramics have been widely used in dentistry because of their ability to provide excellent cosmetic results that mimic natural teeth they exhibit good mechanical strength when subjected to masticatory efforts. This rapid development of ceramic systems and processing enabled the treatment of teeth in both the anterior and posterior areas with primary objective of properly restoring form, function and esthetic excellence without the presence of metal. In 1774, first dental porcelain was fabricated based on “green traditional” porcelain. However these prostheses were abandoned due to their high opacity. In 1838, Elias Wildman made porcelain that was more translucent close to the teeth (2).

But, this feldspathic porcelain showed a great mechanical fragility due to crystalline structure. This lead to the introduction of metal infrastructure associated with ceramics, in order to compensate for the low fracture resistance of the porcelain. This association became known as “metalloceramic prosthesis” and represents a milestone in the technological advancement of dental prostheses. A series of events took place after the introduction of these prostheses which improved the technique of ceramic processing (3).

The use of metalloceramic prostheses over the last 50 years has minimized the problem with porcelain fragility; however its esthetic potential was limited due to the presence of metal. This metal framework acts as a barrier to the transmission of light, giving the prosthetic dental restoration an unesthetic opaque aspect with the presence of darkening at the cervical region of the prosthesis. So to overcome this Aluminium oxide (Al_2_O_3_) as a reinforcing phase in dental porcelain was developed in 1965 (2).

The first ceramic infrastructures made of Alumina were obtained by a process known as craft ceramic infiltration slip casting, where an infrastructure of high density crystal is prepared with a small amount of glass. During sintering, the glass melts and penetrates the infrastructure through capillary action and creates a ceramic surface with a very low porosity and high flexural strength. Three infiltrating system were developed reinforcing with alumina and strengthening with Magnesia or Zirconia. The flexural strength varies according to the reinforcement used. Alumina (400 Mpa), Magnesia (300 Mpa) and Zirconia (750 Mpa).However, both the concentration of alumina and Zirconia shown in these ceramic systems impart improvement of the optical qualities of the restoration, due to its high opacity (4, 5).

Additionally, the porosity incorporated during the manufacture of the infrastructure can affect the strength of these restorations (6).

Parallel to the introduction of infiltrated ceramics, glass ceramics have been improved to be applied in the vacuum injection technique, similar to the traditional technique of metal casting. Two glass ceramic compositions were introduced Leucite based and Lithium disilicate based. The development of new high strength dental ceramics, which appear to be less brittle, less limited in their tensile strength and less subject to time dependant stress failure, has dominated in the later part of 20th century (4, 5).

Zirconia was discovered by the chemist Martin Heinrich Klaproth in Germany in 1789 and was isolated by the Swedish chemist Jons Jacob Berzelius in 1824 (7).

It is produced though a series of steps that separate the ZrO2 and the impurities from the ore (ZrSiO_4_). ZrO_2_ ceramics have three different crystallographic forms depending on temperature. At room temperature and up to 1170°C the material is in the monoclinic phase (M). Over this temperature and up to 2370°C the material transforms to the tetragonal phase (T) and then to the cubic phase (C) at yet higher temperatures (3).

The transformation of the ZrO_2_ material from tetragonal to monoclinic phase is combined with 3-4% volumetric expansion.7 It is this transformation of ZrO_2_ from the monoclinic to the tetragonal phase that differentiates it from other ceramic materials.

The addition of oxides of Calcium, Magnesium, Cerium, Ytrium to pure ZrO_2_ can create partially stabilized zirconia (PSZ) which is stable at room temperature. PSZ usually consists of all three phases (7).

The cubic phase is not a favourable phase because it accumulates the yttrium and the remaining tetragonal phase is not stable (7). It is also possible to create PSZ at room temperature consisting of primarily the tetragonal phase (Tetragonal Zirconia Polycrystalline-TZP) by the addition of 2-3% mol yttrium oxide (7).

The ability to retain the tetragonal phase at room temperature provides very favorable mechanical properties. Under stress, i.e., at the tip of a crack, the 3Y-TZP undergoes a phase transformation from tetragonal to monoclinic phase. This phase transformation results in a 3-4% volumetric expansion inducing a compressive stress in the area of the crack and theoretically prevents crack propagation (3).

This strengthening mechanism is known as transformation toughening and makes ZrO_2_ much stronger compared to all other ceramic materials.

Inspite of improved mechanical properties, acceptable marginal adaptation and biocompatibility the whitish opacity of zirconia is an obvious esthetic disadvantage. The zirconia framework is often veneered with conventional feldspathic porcelain to achieve a natural appearance. However it is difficult to achieve sufficient bond strength between zirconia and the veneering material. Achieving sufficient bond strength between the veneering ceramic and the zirconia core is a major challenge in the long term clinical success of veneered zirconia restorations. After the fabrication of the zirconia core, surface treatments such as airborne-particle abrasion are typically performed on the zirconia surface before veneering. Heat treatment is recommended after airborne particle abrasion in dental practice in order to release the compressive stresses initiated by the air borne particle abrasion. The bonding between the zirconia core and the tooth is also important for the clinical success of zirconia restorations. Attempts have been made to create roughened ceramic surface to provide higher bond strength, thus creating a stronger micromechanical interlock. To increase the surface area for micromechanical bonding, airborne-particle abrasion has been used. Some reports have suggested that zirconia treated with airborne particle abrasion easily develops surface cracks or fractures, and this may reduce its mechanical strength. However, the sintering conditions are important because they affect the crystal size, the mechanical properties and the stability of zirconia.

A follow up study from OlssonK G *et al* showed that Processing at a proper pre-sintering temperature of zirconia is an important factor because this parameter influences hardness, machinability and roughness of the blocks. Hardness and machinability properties act as reverse factors; this means if hardness of block is adequate, manipulation of blocks is performed safely, but, high hardness is unsuitable for machinability. Moreover, an increase in pre-sintering temperatures generates rougher surfaces (49).

Meanwhile, the most important problem in soft-machining is the difference in the sintering shrinkage of the framework and the enlargement values (4). The major disadvantage of the hard machining technique is more time-consuming and requires very tough and wear-resistant cutting devices. Provision of these pieces of equipment renders the production procedure more costly (50).

Milling zirconia blocks at thin sections provides various types of surface microcrack and defects. It seems that factors such as the grain size of the diamond burs or the rotation speed are effective (51). Surface treatments provide more roughness but decrease toughness and the strength resulting in the exposure of the processing defects to moisture. It has been reported that it induces aging potential and decreases serviceability of the restoration (52).

Ban S and Sato *et al* in their study concluded that, residual stress due to processing the restoration, especially coefficient of thermal expansion (CTE) difference between the fired zirconia and the veneering material, plays a more important role than surface treatments in aging potential (53).

One problem of zirconia application is its adhesion to different substrates. Routine methods for bonding of restorations to hard tooth structures and restorative materials do not provide desired bond strength for ZrO_2_ components (45). Surface treatment of zirconia produces an activated surface in different applications (46). Conventional surface treatment techniques are 1) acid etching (typically Hydrofloric acid), 2) abrasion with diamond (or other) rotary instruments, 3) air abrasion with alumina (or other particles, 4), application of different laser types and 5) a combination of these techniques that actually roughen surfaces.

Since zirconia is resistant to aggressive chemical treatment, very aggressive mechanical abrasion methods must be used to provide sufficient surface roughness (47).

Zandparsa *et al* compared the effect of airborne particle abrasion, acid-etching (Piranha solution), and application of an alloy primer on shear bond strength of zirconia to enamel and concluded that airborne particle abrasion in combination with the application of a zirconia primer provides a durable bond strength. Surface grinding is a commonly used alternative for roughening the surface of ZrO_2_ to improve mechanical bonding (54). There are several methods used for surface roughening: roughening with abrasive paper or wheels (SiC or Al_2_O_3_), particle air-abrasion using Al2O3 or other abrasive particles ranging in size from 50 to 250 μm and grinding with a diamond bur. A novel surface roughening technique that has been explored for ZrO_2_ is selective infiltration etching (SIE). SIE uses a heat-induced maturation process to pre-stress surface grain boundaries in ZrO_2_ to allow infiltration of boundaries with molten glass.

The use of SIE improved the nano-mechanical retention of zirconia by increasing the surface area available for bonding. This was confirmed by atomic force microscopy work carried out by Casucci *et al*, showing that the surface roughness of ZrO_2_ is significantly greater after SIE, when compared to particle air-abrasion or HF etching (54, 55). AFM has shown that the application of hot chemical etching solution produces a surface roughness that is significantly greater than SIE. Recently, use of lasers is a method for roughening of surface of zirconia restorations. Results of laser-based studies are controversial.

Akyl *et al* reported that roughening of the surface of Y-TZP ceramic by Er:YAG laser increased the shear bond strengths of ceramic to dentin and reduced microleakage scores (55). It has been shown that the Nd:YAG laser created more roughness on zirconia when compared to the CO_2_ laser and abrasion treatments. Silica-coating increased the SBS of lased and non-lased zirconia. Significant microcracks were found on specimens treated with CO_2_ (56).

A study conducted by Kosmac T *et al* to evaluate the effect of grinding and sandblasting on the microstructure, biaxial flexural strength and reliability of two yytria stabilized tetragonal zirconia (Y-TZP) ceramics showed that sandblasting may provide a powerful technique for strengthening Y-TZP in clinical practice (17).

Jin Young Song *et al* investigated the effect of airborne particle abrasion and heat treatment on the microstructure, biaxial flexural strength and reliability of Y-TZP zirconia ceramics before veneering and cementation. Result showed that the group with airborne particle abraded lower surfaces showed significantly higher flexural strength than the untreated group. SEM images showed rough and irregular surfaces on the airborne particle abraded surface (44).

Ozcan M *et al* evaluated the effect of air particle abrasion protocols on the biaxial flexural strength, surface characteristics and phase transformation of zirconia after cyclic loading. It showed that air abrasion protocols increased the roughness and monoclinic phase, but in turn abrasion with 30 μm alumina particles coated with silica has increased the biaxial flexural strength of the tested zirconia (45).

Eighty disks each of two commercially available Ceramill (AMANNGIRRBACH) and ZR-White (UPCERA) Y-TZP were divided into 10 groups of 8 specimens each, and then heat treatment after airborne-particle abrasion and air borne particle abrasion alone were applied to the upper (veneering) and lower (cementing) surfaces of the specimens.

The airborne particle abrasion used was 50 μm alumina and 50 μm silica coated alumina particles under pressure 30 psi for 15 seconds at a direction perpendicular to the surface with an airborne-particle abrasion device for the specimens in the airborne-particle abraded groups 2, 3, 6, 7, 8, 9 and 10.

Heat treatments were performed at a starting temperature of 500ºC, heating rate of 100°C/min ending temperature of 1000ºC and 15 minutes holding time without vacuum for the specimens in the group 4, 5, 9 and 10.

The specimens were cleaned for 15 minutes in an ultrasonic bath containing distilled water.

To determine the fracture strength load, a disc of 10mm diameter was used to place 3 hardened steel balls of 3mm diameter separated each other by 120 degrees (described in the ISO standard 6872 for dental ceramics). Each specimen was centrally placed on this disc. The lower surface mimicking the internal surface of zirconia was the tension side facing the supporting device testing, while the upper surface mimicking the external surface of the zirconia core was loaded with a flat punch (1 mm in diameter). A universal testing machine was used to determine the fracture strength.

According to the findings of this study, the mean fracture strength of commercially available AMANNGIRRBACH Zirconia showed a significant higher value compared to the UPCERA Zirconia (P value < 0.001), Airborne abrasion treatment to the specimens showed a significant difference between the abraded groups and the control group; further AMANNGIRRBACH specimens gave a higher value compare to the UPCERA specimens. The study also revealed that the heat treatment of the specimens increases the fracture strength compared to the control group, but heat treatment following the air abrasion reduces the fracture strength of the sample than the air abraded group.

Restorations using Zirconia ceramics are processed through a series of steps including cutting, polishing, airborne particle abrasion, heat treatment and firing of the veneering porcelain. To achieve successful outcomes for Zirconia based restorations, each step with an effect on the properties should be thoroughly studied.

In the present study, two surfaces of the specimens were divided into the upper and lower surface. Airborne particle abrasion of the upper and lower surface and heat treatment of the upper surface following air abrasion were applied to mimic the preparations for veneering and cementation. The upper surfaces were facing the loading piston, and the lower surfaces were facing the supporting device during the fracture strength test.

Lawn *et al* reported that tensile stresses are distributed on the inner surface of the crown. Therefore, the lower surfaces were placed on the tension side in this study. Guzzato *et al* also stated that, the mechanical property of Zirconia specimens were dependent on the amount of monoclinic ZrO_2_ content of the lower surface, over which the tensile stress was distributed (44).

The transformed monoclinic phase, which was induced by air borne abrasion creates a layer of compressive stress which counteracts the strength degradation caused by the airborne-particle abrasion induced flaws. The mechanical properties of Zirconia are strongly affected by the stress induced transformation of the surface over which tensile stress is distributed.

Swain *et al* reported that transformation from tetragonal to monoclinic phase causes approximately 4% volume expansion and plastic deformation, there by forming compressive stress on the surface leading to high fracture toughness. Although the thickness of the compressive surface layer formed during airborne particle abrasion is small, it is effective in increasing the strength of the Y-TZP Zirconia. The length of surface flaws which are introduced by air abrasion does not seem to exceed significantly the thickness of the compressive surface layer. This indicates that the fracture strength of Zirconia is affected more by increased fracture toughness due to transformation toughening than due to flaws introduced by airborne particle abrasion.

Denry *et al* reported that heat treatment after mechanical surface treatment reduced the size of the flaw and blunted it thereby reducing the stress at the crack tip, which is related to the increase in the fracture strength (16).

However, Swain *et al* reported that heat treatment of the specimens was accompanied by the reverse monoclinic to tetragonal phase transformation and negligible amount of monoclinic phase was detected after heat treatment. It is thus concluded that compressive stress was released and the flexural strength of the airborne particle abraded specimen dropped after heat treatment. The study also showed that monoclinic content disappeared after the heat treatment. Some of the laboratory or clinical procedures on a Zirconia core, for example air abrasion, which is used to increase bond strength between the veneering ceramic and the core to increase the adhesion of the cement, may also be used to increase flexural strength by promoting phase transformation. However, microcracking and strength degradation may result from an excess amount of transformed monoclinic phase and slow crack growth (44).

Therefore, further studies are needed to investigate the fatigue behaviour of the dental Y-TZP ceramics under clinical conditions, where the material is exposed to thermaland mechanical cycling in a chemically active environment over a long period of time.

## CONCLUSION

The present in vitro study was conducted to evaluate the effect of airborne particle abrasion using different types of alumina abrasive particles and heat treatment on the flexural strength of two commercially available zirconia before veneering and cementation. Eighty disks each of commercially available Zirconia ZR WHITE (UPCERA) & Ceramill white (AMMANNGIRRBACH) Pre sintered Zirconia block of size (15.2+/-0.03 mm in diameter & 1.2+/- mm thick were fabricated. After different surface treatment with heat and different silica particle sizes, the specimens were subjected to fracture strength test using universal testing machine. (MODEL LR50K; INSTRON CORP, LLOYD INSTRUMENTS). The readings were then subjected to statistical analysis. From the results obtained the following conclusions can be made. The* in vitro* fracture strength of zirconia specimens treated with an airborne abrasion both on the veneering surface (50 µm silica coated Al_2_O_3_) and the cementing surface (50 µm Al_2_O_3_) was significantly higher than the heat treated and the control group. Airborne particle abrasion followed by the heat treatment reduces the fracture strength of the specimen than that of the group treated only by the air abrasives.

The fracture strength of a commercially available Ceramill (AMANNGIRRBACH) is greater than that of a Zirconia from ZR-White (UPCERA) variety.

## SUMMARY

In recent years, Zirconia restorations have become popular due to the increased interest in esthetic and mechanical property. The success of a Zirconia restoration largely depends on its

resistance to the fracture load, method of processing of the Zirconia material (soft machining/hard machining ,surface treatments) and also the patient selection .Keeping this in mind, the present study was undertaken to recognize the effect of various surface treatments on the fracture strength of the two commercially available Zirconia.

Two commercially available pre sintered yttrium stabilized Zirconia blanks ZR WHITE (UPCERA) & Ceramill (AMMANGIRRBACH) are used to produce the disc shaped specimens of size (15.2 ± 0.03 mm in diameter and 1.2 ± 0.03 mm thick) from each Zirconia blank. All disc shaped specimens are heated at 1200°C in a furnace for 2 hours to form homogenous tetragonal ZrO. the dimensions of the specimen are measured with digital caliper. The thickness and the diameter of each specimen are calculated as the means of 3 measurements made at random sites.80 discs from each zirconia blank are divided into ten groups of 8 specimens each heat treatment after airborne particle abrasion using 50 µm Al_2_O_3_.

Particle and 50 µm silica coated Al2O3 and abrasion from 2 airborne particles alone are applied to the upper and lower surface of the specimens. Each specimen is held under pressure of 30 psi for 15 seconds at a direction perpendicular to the surface and at a distance of 30 mm with an airborne particle abrasion device for specimens in the airborne particle abraded groups. Heat treatments were performed at a starting temperature of 500°C heating rate of 100°C/min, ending temperature of 1000°C and 15 minutes holding time without vacuum for the specimens in the group 4, 5, 9 and 10. Airborne particle abrasion mimicking the preparation for cementation was applied to the lower surfaces with 50 µm alumina and silica coated alumina particles for the specimens in the groups 6, 7, 8, 9 and 10.The specimens were cleaned for 15 minutes in an ultrasonic bath containing distilled water.

To determine the fracture strength load, a disc of 10mm diameter was used to place 3 hardened steel balls of 3 mm diameter separated each other by 120 degrees (described in the ISO standard 6872 for dental ceramics). Each specimen was centrally placed on this disc. The lower surface mimicking the internal surface of zirconia was the tension side facing the supporting device testing, while the upper surface mimicking the external surface of the zirconia core was loaded with a flat punch (1mm in diameter). A universal testing machine was used to perform the test at a cross head speed of 1 mm/min. The failure stress was calculated with the equation listed in ISO 6872. The results were then statistically analysed. A post hoc test was used for pair wise comparisons.

The mean fracture strength of commercially available Ceramill (AMMANGIRRBACH) Zirconia showed a significant higher value compared to the ZR WHITE (UPCERA) Zirconia *P*<0.001, airborne abrasion treatment to the specimen showed a significant difference between the abraded groups and the control group (*P*<0.001) FURTHER Ceramill (AMMANGIRRBACH) specimens gave a higher value compare to the ZR WHITE (UPCERA) Specimens. The study also reveal that the heat treatment of the specimens with the significant value (*P*<0.001) compared to the control group, but heat treatment following the air abrasion reduces the fracture strength of the sample than the air abraded group. Knowledge of such parameters enables the clinician to make appropriate choice in various clinical situations.
